# Association of Hematological and Biochemical Parameters and HLA-DRB1 Alleles With Anti-cyclic Citrullinated Peptide Autoantibodies in Sudanese Rheumatic Patients

**DOI:** 10.7759/cureus.58551

**Published:** 2024-04-18

**Authors:** Khalid E Khalid

**Affiliations:** 1 Department of Basic Medical Sciences, Faculty of Applied Medicla Sciences, Al-Baha University, Al-Baha, SAU

**Keywords:** biochemical parameters, hematological parameters, acpa, hla class ii genotype, rheumatoid arthritis

## Abstract

Introduction

Anti-citrullinated protein/peptide antibodies (ACPA) are crucial for the diagnosis and prognosis of rheumatoid arthritis (RA) and are associated with class II HLA-DRB1 alleles.

The study’s goal was to determine how DRB1 alleles and hematological and biochemical parameters affect ACPA production in RA patients from Sudan.

Methods

The study analyzed the hematological and biochemical parameters and the frequency of HLA-DRB1 alleles in 120 RA patients and 100 controls. Automated analyzers, ELISA, the latex agglutination test, and the Westergren method were utilized for hematological and biochemical testing. HLA class II alleles were genotyped using polymerase chain reaction-sequence-specific primers (PCR-SSP). The student’s t-test and the chi-square (*Χ*^2^) test were employed to identify significant alterations between the examined parameters and allele frequencies.

Results

A total of 51.7% of 120 RA patients tested positive for ACPA (ACPA+). Among those patients, the DRB1*04 and *10 alleles were significantly more prevalent (22.2% vs. 8.9%, P = 0.048 and 23.8% vs. 8.9%, P = 0.030, respectively).

RA patients had significantly higher counts of platelet count test (PLT; P = 0.011), lymphocytes (LY; P = 0.000), neutrophils (NE; P = 0.025), monocytes (MO; P = 0.000), eosinophils (EO; P = 0.000), neutrophil-to-lymphocyte ratio (NLR; P = 0.006), C-reactive protein (CRP; P = 0.000), and erythrocyte sedimentation rate (ESR; P = 0.000) than controls. Patients also showed low counts of red blood cells (RBC; P = 0.003), hemoglobin (Hb; P = 0.024), mean platelet volume (MPV; P = 0.000), and basophils (BA; P = 0.048). ACPA+ RA patients had elevated white blood cells (WBC; P = 0.046), PLT (P = 0.029), and low mean corpuscular hemoglobin concentration (MCHC; P = 0.022). The hematological and biochemical parameters of ACPA+ RA patients with the DRB1*04 or *10 alleles did not differ significantly.

Conclusions

We found significant differences in hematological and biochemical parameters between RA patients and controls that had nothing to do with ACPA positivity or the frequency of DRB1*04 or *10 alleles.

## Introduction

Rheumatoid arthritis (RA) is a persistent inflammatory disease that primarily affects tissues and organs, particularly the joints, leading to chronic inflammation of the synovial membrane and destruction of bone tissue [1]. Because of RA’s inflammatory and systemic characteristics, the majority of affected patients experience a progressive pattern of disease activity that fluctuates in intensity over time. It is commonly acknowledged in clinical practice that effectively and swiftly controlling rheumatoid inflammation is vital for ensuring the safety of patients. This control should also be sustained for as long as possible [2].

It is important to regularly and consistently evaluate the severity of rheumatoid inflammation in clinical settings using quantitative methods. There are laboratory tests that can complement clinical practice and X-rays to improve the accuracy of RA diagnosis.

These tests encompass hematological measures, such as red blood cells (RBC), hemoglobin (Hb), and hematocrit (HCT), along with biochemical inflammatory markers, such as anti-citrullinated protein/peptide antibodies (ACPA), rheumatoid factor (RF), erythrocyte sedimentation rate (ESR), and C-reactive protein (CRP). This is due to the marked alteration of these parameters during inflammation in RA. Even though numerous studies have shown that ACPA characterizes patients with a poor prognosis in terms of joint injury and inflammation [3,4], many authors have reported that the hematological and biochemical markers are associated with RA disease activity [5-9].

There is a growing body of research indicating that patients with RA can be categorized into two main groups depending on their ACPA status: ACPA-positive RA (also known as ACPA+ RA) and ACPA-negative RA (also known as ACPA− RA). These groups possess various genetic and environmental risk factors that are associated with different disease entities. Studies suggest that those with ACPA− RA who experience arthralgia initially tend to have joints that are more sensitive, experience greater difficulty in clenching their fists, and have a shorter duration of symptoms compared to those with ACPA+ RA. However, ACPA+ RA progressed to arthritis more quickly after that [10]. Interestingly, human leukocyte antigen (HLA) class II molecules make up most of the genetic risk factors in RA patients who test positive for ACPA [11]. Several studies that looked at HLA alleles and genotypes in ACPA+ RA patients found that certain subgroups of HLA-DRB1 polymorphisms, such as HLA-DRB1*01, *04, and *10, are linked to the severity of the disease’s structural damage. These polymorphisms are also likely to influence the production of ACPA by affecting antigen presentation [12-14]. This is the first study to look at the link between ACPA and hematological and biochemical markers, as well as the frequencies of HLA-DRB1 risk alleles in our RA population.

## Materials and methods

Study subjects

This cross-sectional study was conducted over two years (September 2020 to October 2022) at the rheumatology departments of two tertiary hospitals in Khartoum State, Sudan. The study included 120 individuals who were clinically diagnosed with RA by board-certified rheumatologists in accordance with the American College of Rheumatology guidelines [15].

Patients with autoimmune inflammatory diseases, chronic diseases, hematologic diseases, infections, cancers, aplastic anemia, or taking steroids, antiplatelets, anticoagulants, or disease-modifying anti-rheumatic drugs (DMARDs) were excluded from this study. This is because these conditions and their treatment regimens may have an impact on the hematological and biochemical parameters that are relevant to our study, regardless of the severity of RA disease activity.

The clinical interview form collects information such as age, sex, clinical data, and laboratory profiles. After clinical and laboratory testing, 100 healthy individuals (controls) who were matched for age and gender were selected from the same hospitals. Written consent was obtained from study participants after they were informed of the research goals and intentions. The study received clearance from the University of Gezira’s research ethics committee (ref. no. UGIREC/2020) and was conducted in accordance with the Helsinki Declaration.

Blood sampling and laboratory tests

Ten milliliters of blood were collected from study participants in order to conduct hematological assays, such as complete blood count (CBC) and differential blood count (DBC), and to assess biochemical inflammatory indicators, such as ACPA, CRP, and ESR. The CBC tests were for HCT, PLT, Hb, WBC, and RBC. Some more constants related to erythrocytes were assessed, including mean cell hemoglobin volume (MCV), mean cell hemoglobin (MCH), and mean cell hemoglobin concentration (MCHC). The DBC contained the percentages of various blood cell types, including neutrophils (NE), monocytes (MO), eosinophils (EO), basophils (BA), and lymphocytes (LY). First, we placed 3 mL of blood into a vacuum tube without an anticoagulant to measure ACPA and CRP. Subsequently, 1.8 mL of blood was collected into a vacuum tube containing sodium citrate to measure the ESR, and another 3 mL into a vacuum tube containing ethylenediaminetetraacetic acid (EDTA) for the hematological assays. 

Biochemical and hematological analysis

We assessed the ACPA using an enzyme-linked immunosorbent test (ELISA; Euro Diagnostic, Sweden). We considered it positive when the ACPA concentration was >25 U/mL. The RF for IgM was measured in patients’ sera by a rapid latex agglutination test (NS Bio-Tec, Egypt). Serum CRP was quantified using ELISA (Roche/Hitachi analyzer, Basel, Switzerland), defining the normal range as 5-20 IU/mL. ESR was further determined using the traditional Westergren method [16]. The automated Sysmex XP-300A hematology analyzer (Sysmex Corporation, Kobe, Japan) was used to measure blood counts as per instructions provided by the manufacturer.

HLA-DRB1 genotyping 

The QIAamp® DNA Micro Kit (Qiagen, CA, USA) was used to isolate DNA from whole blood. HLA class II DRB1 genotyping was conducted via sequence-specific primers (SSP) and polymerase chain reaction (PCR) with low-resolution kits (ROSE, Germany). We followed the guidelines provided by ThermoFisher, UK, when using an Applied Biosystems 9700 thermocycler to amplify DNA. Ten milliliters of the PCR master mix comprised 50 ng of target DNA, Taq DNA polymerase (5 U/µL), water, and PCR mix, which included PCR buffer, glycerol, cresol red, and detergents. The PCR amplifications were carried out in 24-well plates containing the dried DR primers and dNTP-Mixes using an Applied Biosystems 9700 thermocycler (ThermoFisher, UK). The SSP-PCR program was carried out as follows: an initial cycle at 94°C for one minute, followed by 10 cycles at 94°C for 10 seconds and 65°C for one minute, then 20 cycles at 94°C for 10 seconds, 61°C for 50 seconds, and 72°C for 30 seconds, a final extension cycle at 72°C for two minutes, and finally a cycle at 4°C. A volume of 10 mL of the PCR products was loaded onto a 2% agarose gel, separated by electrophoresis, and then seen under ultraviolet light using a gel documentation system. The ROSE company provided the HLA-DRB typing software.

Statistical analysis

The statistical studies were conducted using the IBM SPSS Statistics, version 23.0 (IBM Corp., Armonk, NY) application for Mac OS X. All continuous variables had their means, standard deviations (SD), and standard errors of the means (SEM) calculated. The categorical data were presented using counts, frequencies, and percentages. The unpaired student’s t-test was used to compare the significantly different hematological and biochemical parameters between the patient and control groupings. We used the chi-square (*Χ*^2^) test to find out if there was a statistically significant difference in the frequency of HLA-DRB1 alleles between study groups. A two-tailed P-value of less than 0.05 was required to establish a significant connection for each test.

## Results

The patients’ in-hospital clinical features and laboratory results are listed in Table 1. The mean ACPA, CRP, and ESR values did not differ significantly between the male and female patients.

**Table 1:** Basic characteristics of study subjects NA, not available; M/F, male/female; n, number; SD, standard deviation; ACPA, anti-citrullinated protein/peptide antibodies; CRP, C-reactive protein; ESR, erythrocyte sedimentation rate

Demographic and lab results	Patients (n = 120) (mean ± SD)		Controls (n = 100) (mean ± SD)	
	M (n = 16)	F (n = 104)	M (n = 11)	F (n = 89)
Age/years	44.81 ± 14.65	44.83 ± 14.06	38.55 ± 14.37	43.62 ± 9.89
Disease duration/years	5.19 ± 2.23	0.62 ± 0.44	NA	NA
ACPA (U/mL)	593.75 ± 165.60	605.35 ± 90.74	NA	NA
CRP (mg/L)	16.70 ± 6.94	15.74 ± 1.82	NA	NA
ESR (mm/h)	49.94 ± 35.91	67.84 ± 26.73	NA	NA

As shown in Table 2, half (51.7%) of the RA patients tested were positive for ACPA and CRP. Morning stiffness is reported in 68.0% of RA cases, according to the clinical picture. Among the patients, ulnar deviation was the most prevalent deformity, and the most affected joints were the hands, knees, and wrists.

**Table 2:** Clinical characteristics of study cases M/F, male/female; n, number; %, percentage; ACPA, anti-citrullinated protein/peptide antibodies; RF, rheumatoid factor; CRP, C-reactive protein

Clinical characteristics		RA patients (n = 120)			
		Yes (%)		No (%)	
		M	F	M	F
Morning stiffness		11	70	5	34
ACPA+		9	53	7	51
RF+		3	25	13	79
CRP+		11	51	5	53
Joints affected by RA	Fingers	11	37	5	13
	Hands	12	89	4	15
	Wrists	15	93	1	11
	Elbows	11	65	5	39
	Shoulders	11	61	5	43
	Neck	2	36	14	68
	Back	5	26	11	78
	Toes	7	55	9	49
	Feet	8	69	8	35
	Ankles	11	75	5	29
	Knees	12	90	4	14
	Hips	4	17	12	87
Deformities	Boutonniere deformity	2	14	14	90
	Ulnar deviation	3	27	13	77
	Swan-neck deformity	1	16	15	90
	Z thumb	3	14	13	92
	Presence of rheumatoid nodules	0	8	16	98

Although DRB1*08 was considerably lower (4.8% vs. 24.6%, P = 0.003), the allele frequencies of DRB1*04 and *10 were significantly higher in ACPA+ RA patients (22.2% vs. 8.9%, P = 0.048 and 23.8% vs. 8.9%, P = 0.030, respectively) (Table 3). 

**Table 3:** HLA-DRB1 allele frequency between controls and RA subgroups AF, allele frequency; n, number of individuals; RA, rheumatoid arthritis; ACPA, anti-citrullinated protein/peptide antibodies *P-values <0.05 were significant (bold).

Allele HLA-DRB1	Controls (n = 194)		RA patients (n = 234)		RA patient subgroups				Patients vs. controls	ACPA+ vs. ACPA-
					ACPA+ (n = 63)		ACPA- (n = 57)			
	n	AF	n	AF	n	AF	n	AF	P-value	
*01	13	6.7	13	5.5	6	9.5	05	8.9	0.687	1.000
*03	20	10.3	23	9.7	09	14.3	07	12.5	0.873	0.794
*04	10	5.1	22	9.3	14	22.2	05	8.9	0.101	0.048*
*07	23	11.8	12	5.1	04	6.3	05	8.9	0.013*	1.000
*08	19	9.7	27	11.4	03	4.8	14	23.3	0.639	0.003*
*09	02	1.0	01	0.4	01	1.6	0	0	1.000	1.000
*10	16	8.2	33	14.0	15	23.8	05	8.9	0.067	0.030*
*11	24	12.3	26	11.0	02	3.2	07	12.5	0.763	0.084
*13	46	23.6	49	20.8	04	6.3	05	8.9	0.559	1.000
*15	21	10.8	28	11.9	05	7.9	04	7.2	0.762	1.000

There was a statistically significant difference between the control group and the RA patients in terms of CRP and ESR levels (P = 0.000 for both). Additionally, RA patients had significantly greater levels of the hematological PLT (P = 0.011), LY (P = 0.000), NE (P = 0.025), MO (P = 0.000), EO (P = 0.000), NLR (P = 0.006), and PLR (P = 0.000) when contrasted with the control group. On the other hand, the patient’s hematological tests revealed significantly reduced levels of RBCs (P = 0.003), Hb (P = 0.024), MPV (P = 0.000), and BA (P = 0.048) (Table 4).

**Table 4:** Hematological and biochemical parameters in patients and controls SEM, standard error of the mean; n, number; CI, confidence intervals; WBC, white blood cells; RBC, red blood cells; Hb, hemoglobin; HCT, hematocrit; MCV, mean corpuscular volume; MCH, mean corpuscular hemoglobin; MCHC, mean corpuscular hemoglobin concentration; PLT, platelet count test; MPV, mean platelet volume; LY, lymphocytes; NE, neutrophils; MO, monocytes; EO, eosinophils; BA, basophils; NLR, neutrophil-to-lymphocyte ratio; PLR, platelet-to-lymphocyte ratio; ACPA, anti-citrullinated protein/peptide antibodies; CRP, C-reactive protein; ESR, erythrocyte sedimentation rate *P-values <0.05 were significant (bold).

Parameter	Controls (n = 100) (mean ± SEM)	RA patients (n = 120) (mean ± SEM)	P-value	95% CI
WBC (10^3^/mL)	6.14 ± 0.13	6.33 ± 0.18	0.523	-0.65 to 0.33
RBC (10^6^/mL)	4.73 ± 0.04	4.54 ± 0.05	0.003*	-0.06 to 0.32
Hb (g/dL)	13.39 ± 0.14	12.35 ± 0.12	0.024*	-0.21 to 0.53
HCT (%)	40.44 ± 0.34	39.48 ± 0.45	0.085	-0.13 to 2.05
MCV (fL)	87.10 ± 0.64	85.68 ± 0.62	0.118	-3.19 to 0.36
MCH (pg)	27.71 ± 0.58	26.63 ± 0.25	0.076	-2.26 to 0.11
MCHC (g/dL)	31.77 ± 0.63	31.00 ± 0.11	0.191	-1.92 to 0.39
PLT (cell/cmm)	251.15 ± 7.44	276.16 ± 6.33	0.011*	-5.89 to 44.14
MPV (fL)	9.31 ± 0.92	8.03 ± 0.84	0.000*	-0.72 to 0.83
LY (%)	32.34 ± 0.93	37.60 ± 0.91	0.000*	-2.66 to 7.85
NE (%)	53.54 ± 1.03	56.96 ± 1.08	0.025*	0.43 to 6.40
MO (%)	8.21 ± 0.31	9.56 ± 0.26	0.000*	0.55 to 2.14
EO (%)	0.81 ± 0.39	0.80 ± 0.86	0.000*	-0.07 to 0.34
BA (%)	0.18 ± 0.04	0.09 ± 0.03	0.048*	0.18 to 0.00
NLR	1.63 ± 1.04	2.24 ± 2.00	0.006*	1.74 to 2.19
PLR	7.21 ± 3.23	10.12 ± 6.79	0.000*	8.06 to 9.56
CRP (mg/L)	7.92 ± 0.79	16.31 ± 1.83	0.000*	-18.39 to 10.42
ESR (mm/h)	28.59 ± 0.97	66.02 ± 2.62	0.000*	-43.38 to 31.47

A comparison between ACPA+ and ACPA− RA patients revealed significantly higher WBC and PLT counts, as well as lower MCHC (P = 0.022) levels. Both the DBC and the biochemical markers’ high levels fell short of a meaningful threshold (Table 5).

**Table 5:** Hematological and biochemical parameters in ACPA+/- RA patients SD, standard deviation; n, number; CI, confidence intervals; WBC, white blood cells; RBC, red blood cells; Hb, hemoglobin; HCT, hematocrit; MCV, mean corpuscular volume; MCH, mean corpuscular hemoglobin; MCHC, mean corpuscular hemoglobin concentration; PLT, platelet count test; MPV, mean platelet volume; LY, lymphocytes; NE, neutrophils; MO, monocytes; EO, eosinophils; BA, basophils; NLR, neutrophil-to-lymphocyte ratio; PLR, platelet-to-lymphocyte ratio; ACPA, anti-citrullinated protein/peptide antibodies; CRP, C-reactive protein; ESR, erythrocyte sedimentation rate *P-values <0.05 were significant (bold).

Parameter	RA patients (n= 120) (Mean±SEM)		P-value	95% CI
	ACPA+ N = 63	ACPA- N = 57		
WBC (10^3^/mL)	6.53 ± 0.25	5.83 ± 0.22	0.046*	-1.36 to -0.054
RBC (10^6^/mL)	4.73 ± 0.05	4.74±0.06	0.899	-0.17 to 0.15
Hb (g/dL)	12.43±0.18	12.67±0.17	0.332	-0.22 to 0.76
HCT (%)	40.40±3.78	40.49±3.70	0.902	-1.18 to 1.50
MCV (fL)	85.54±0.50	85.81±0.46	0.883	-2.22 to 2.75
MCH (pg)	26.38±0.41	26.86±0.31	0.376	-0.52 to 1.49
MCHC (g/dL)	30.73±0.18	31.26±0.14	0.022*	0.08 to 0.96
PLT (cell/cmm)	289.84±9.73	261.83±8.23	0.029*	-54.43 to -5.23
MPV (fL)	9.42±0.70	9.53±0.73	0.401	-0.67 to 0.91
LY (%)	33.13±1.49	31.52±1.22	0.402	-5.14 to 2.25
NE (%)	57.41±1.49	56.48±1.66	0.677	-3.60 to 5.02
MO (%)	9.60±0.36	9.55±0.39	0.926	-0.92 to 1.14
EO (%)	0.87±0.11	0.77±0.12	0.508	-0.19 to 0.43
BA (%)	0.11±0.04	0.07±0.03	0.460	-0.06 to 0.14
NLR	2.26±1.75	2.24±2.31	0.957	1.88 to 3.62
PLR	10.63±7.87	9.69±5.86	0.463	8.89 to 11.38
CRP (mg/L)	17.09±2.41	14.66±2.71	0.503	-5.98 to 8.58
ESR (mm/h)	66.89±3.92	64.64±3.54	0.669	-14.24 to 6.54

No statistically significant changes were seen in the hematological and biochemical parameters in ACPA+ RA patients with the DRB1*04 or *10 alleles (Table 6), except for the MCV (P = 0.021).

**Table 6:** Hematological and biochemical parameters in ACPA+/- RA patients with HLA-DRB1*4 and *10 alleles SD, standard deviation; n, number; CI, confidence intervals; WBC, white blood cells; RBC, red blood cells; Hb, hemoglobin; HCT, hematocrit; MCV, mean corpuscular volume; MCH, mean corpuscular hemoglobin; MCHC, mean corpuscular hemoglobin concentration; PLT, platelet count test; MPV, mean platelet volume; LY, lymphocytes; NE, neutrophils; MO, monocytes; EO, eosinophils; BA, basophils; NLR, neutrophil-to-lymphocyte ratio; PLR, platelet-to-lymphocyte ratio; ACPA, anti-citrullinated protein/peptide antibodies; CRP, C-reactive protein; ESR, erythrocyte sedimentation rate *P-values <0.05 were significant (bold).

Parameter	Patients with HLA-DRB1*4 and *10 (n = 41) (mean ± SEM)		P-value	95% CI
	ACPA+ (n = 29)	ACPA- (n = 12)		
WBC (10^3^/mL)	6.51 ± 0.68	5.98 ± 0.35	0.452	-1.93 to 0.87
RBC (10^6^/mL)	4.61 ± 0.14	4.72 ± 0.07	0.450	-0.18 to 0.41
Hb (g/dL)	12.60 ± 0.23	12.84 ± 0.33	0.563	-0.58 to 1.12
HCT (%)	40.10 ± 0.62	41.08 ± 1.32	0.449	-3.57 to 1.61
MCV (fL)	85.12 ± 0.94	89.23 ± 1.39	0.021*	-7.57 to -0.65
MCH (pg)	26.76 ± 0.39	27.98 ± 0.61	0.095	-2.67 to 0.22
MCHC (g/dL)	31.38 ± 0.42	31.38 ± 0.20	0.985	-0.82 to 0.84
PLT (cell/cmm)	270.28 ± 10.84	268.00 ± 21.63	0.917	-41.80 to 46.35
MPV (fL)	8.81 ± 0.69	8.64 ± 0.81	0.499	-0.74 to 0.93
LY (%)	32.28 ± 2.32	29.00 ± 3.39	0.441	-7.06 to 10.08
NE (%)	56.55 ± 2.76	61.33 ± 3.60	0.333	-14.65 to 5.08
MO (%)	9.21 ± 0.41	8.29 ± 0.47	0.686	-1.15 to 173
EO (%)	0.90 ± 0.14	0.67 ± 0.23	0.392	-0.31 to 0.77
BA (%)	0.03 ± 0.03	0.08 ± 0.29	0.521	-0.20 to 0.10
NLR	2.40 ± 2.39	2.21 ± 2.02	0.810	1.81 to 2.76
PLR	13.36 ± 12.01	10.73 ± 7.53	0.487	8.43 to 14.57
CRP (mg/L)	22.65 ± 7.74	16.06 ± 2.67	0.311	-5.98 to 8.58
ESR (mm/h)	81.00 ± 10.98	66.72 ± 5.04	0.182	-35.54 to 6.98

## Discussion

In this study, the RA patients were classified into ACPA+/− groups based on the fact that the ACPA marker was highly specific and proved to be important in RA diagnosis and classification [17]. However, several studies have shown that ACPA characterizes patients with a bad prognosis in terms of joint injury as well as inflammation [3,4]. Also, we investigated the much-studied (with mixed) link between ACPA and HLA class II alleles, most especially HLA-DRB1. Our ACPA positive rate of 51.7% was in line with earlier studies that reported a percentage of up to 55% [18-20]; however, it was substantially lower than other studies that showed a 70% positivity rate in RA patients [21]. Reason being, citrullinated peptides might exhibit themselves as autoantigens in the progression of RA when shared epitope (SE) alleles are present [22].

There was an increase in the levels of the HLA-DRB1*04 and *10 alleles in ACPA+ RA patients, according to our previous findings [12]. This association has been detected with HLA-DRB1*04 on its own [23-25], in combination with HLA-DRB1*10 [26], or with HLA-DRB1*09 [27,28]. A number of HLA-DRB1 alleles have been associated with varying degrees of disease severity, including *01, *04, and *10. These variants (i.e., alleles) probably influence antigen presentation, which in turn affects ACPA synthesis [13,14]. These alleles, which are called SE, can be identified in the third hypervariable area of the HLA-DRB1 chain locus (positions 70-74) and in the short arm of chromosome 6. They are similar in that they both contain the same amino acid sequence in the HLA class II beta chain’s antigen-binding site.

Biochemical CRP and ESR levels were substantially higher in RA patients compared to controls, which was in agreement with earlier studies [5,6,29,30]. It is possible that the elevated CRP, acute phase reactants, and rapid sedimentation seen in RA patients are maintaining their activity process.

We found that the levels of PLT, LY, NE, MO, EO, NLR, and PLR were significantly elevated in RA patients. Consistent with previous research in RA patients [6], our data pointed to the presence of inflammatory processes. In parallel with our observations, earlier research indicated that PLT was involved in the inflammatory process of RA [5,31].

Prior research indicated that NLR and PLR levels in RA patients were substantially greater than in healthy controls [32,33]. These levels can be utilized as a marker of the presence of RA [34] and were also discovered to have an independent correlation with disease activity [29,35,36]. Notably, patients with RA who tested positive for ACPA had significantly higher NLR and PLR compared to those who tested negative [37]. We found that RA patients had substantially reduced levels of RBC, Hb, and MPV compared to controls, which was consistent with previous studies [5,6,38,39]. Multiple studies have linked the presence of anemia with severe structural damage, poor joint function, and increased disease activity in RA patients compared to controls [5-8]. Furthermore, normochromic and normocytic anemia were found in Cameron RA patients [40]. Other data showed significantly reduced levels of RBC, Hb, and MPV in Egyptian RA patients compared to controls [41]. Still, other studies came to different conclusions about RBC and Hb levels between patients and controls, so our findings may be considered contradictory [42].

Regarding the MPV value, our results are in line with previous studies that demonstrated significantly lower MPV levels in individuals with RA and ankylosing spondylitis [29,43]. The absence of acute-phase disease could be indicated by a low MPV value in RA patients with high disease activity, as suggested by the study conducted by Tekeoğlu et al. in 2016 [34]. On the other hand, although RA patients’ MPV values were lower than controls’ [9], they were still not statistically significant [6,44].

Our results also showed that ACPA+ RA patients had low MCHC levels and significantly high mean levels of WBC and PLT, without citing any supporting data. According to previous studies, RA patients who test positive for ACPA have significantly higher average values of NLR and PLR than those who test negative. As a result, these two markers may be associated with a dismal RA prognosis [37]. Previous research has shown that ACPA activates PLT in RA patients through the low-affinity immunoglobulin G receptor (FcγRIIa) [45]. Patients with RA who test positive for ACPA may have an increased number of immune cells, such as white blood cells, which are believed to contribute to joint damage and inflammation. This view necessitates additional research using a large sample size to validate the ideas.

In ACPA+ RA patients with the DRB1*04 or *10 alleles, we did not find any statistically significant variations in hematological and biochemical markers. Our findings indicate that the ACPA-positive status, hematological and biochemical markers, and the existence of the risk genes under investigation are unrelated. Additional research needs to be conducted to confirm this idea.

Our finding indicates that ACPA+ RA patients had lower MCHC and very high WBC and PLT levels, although there is no evidence supporting this claim in the literature. According to Targońska-Stępniak et al. (2020), there appears to be a link between the ACPA markers NLR and PLR and a poor prognosis for RA, as these markers were shown to be significantly greater in patients who tested positive for ACPA compared to those who tested negative [37]. Habets et al.’s 2015 research revealed that ACPA activates PLT in RA patients via the FcRIIa low-affinity immunoglobulin G receptor [45]. One possible explanation for the elevated WBC count in RA patients is that these immune cells may inflame and harm their joints. This impression requires additional research with a large sample size to support the principles.

Our data showed that the hematological and biochemical features of ACPA+ RA patients with the DRB1*04 or *10 alleles were not very different from each other. Our findings also imply that there was no association between the presence of investigated risk alleles and the hematological and biochemical markers in ACPA+ RA patients; further research is necessary to confirm this.

The study’s limitation was that the sample size was too small to draw any firm conclusions about the association between ACPA seropositivity, DRB1*04 or *10 allele frequency, and changes in hematological and biochemical markers. More research with bigger samples is needed to determine these associations in other kinds of Sudanese RA patients with different symptoms. In order to draw a complete picture of RA’s clinical progression, future studies should also look at immunological markers.

## Conclusions

In conclusion, RA had significantly greater DBC (LY, NE, MO, and EO) as well as CRP, ESR PLT, NLR, and PLR values than the controls. Conversely, the patients’ RBC, Hb, and MPV values decreased. Patients who tested positive for ACPA had notably elevated WBC and PLT levels, along with decreased MCHC levels. The hematological and biochemical parameters of ACPA+ RA patients with the DRB1*04 or *10 alleles did not differ significantly.
